# Virtual Reality Simulation for Advanced Infection Control Education in Neonatal Intensive Care Units: Focusing on the Prevention of Central Line-Associated Bloodstream Infections and Ventilator-Associated Infections

**DOI:** 10.3390/healthcare11162296

**Published:** 2023-08-14

**Authors:** Jimin Ryu, Mi Yu

**Affiliations:** 1 Department of Nursing, Gyeongsang National University Hospital, 79 Gangnam-ro, Jinju-daero, Jinju 52727, Republic of Korea; alslgirl77@naver.com; 2 College of Nursing, Institute of Medical Science, Gyeongsang National University, 816 Beongil-15, Jinju-daero, Jinju 52727, Republic of Korea

**Keywords:** infection control, neonatal nursing, simulation training, virtual reality, confidence, empathy

## Abstract

This study examined the effects of a virtual reality simulation for advanced infection control education in neonatal intensive care units (VR_AICENICU) on nurses’ infection control knowledge and performance confidence. We examined nurses’ presence, empathy, and program satisfaction using a non-equivalent control group pre-post design. Nurses were divided into an experimental group (*n* = 20) experiencing VR simulation and routine NICU practice and a control group (*n* = 20) with routine NICU practice. The VR_AICENICU program comprised three scenarios: high-risk medication with lipid solution, dressing and management for peripheral inserted central line, and aspiration prevention and skincare management during ventilator use for premature infants. Data were collected between February and July 2022. The experimental group showed significantly greater improvements in infection control knowledge and performance confidence compared to the pre-test. The average scores of presence, empathy, and program satisfaction of the experimental group were 4.39 ± 0.36, 4.33 ± 0.75, and 4.90 ± 0.31, respectively. The VR_AICENICU program has implications for the education needs of nurses working in NICUs and enhances their knowledge and performance of infection control. Future studies should apply the VR_AICENICU program to different severity grades of NICU patients and develop additional VR programs.

## 1. Introduction

Healthcare-associated infections (HAIs) or nosocomial infections are among the major causes of mortality and morbidity in newborn infants admitted to the neonatal intensive care unit (NICU). Considering the incident of newborn deaths from NICU infections in South Korea in 2018, operating a NICU infection control system has been made mandatory nationwide since 2019 [1,2]. Among many infection sources, the incidence of blood-proven sepsis varies widely among NICUs but is generally high, especially in preterm infants because they usually have not only immunological immaturity but also frequent use of invasive procedures and prolonged hospitalization [3]. In the US, sepsis developed in 21–43% of low-birth-weight infants [4]. Additionally, the incidence of blood proven sepsis was 8% in Japan [5] and 21.1% in South Korea [6]. Central lines and noninvasive ventilators, such as a nasal continuous positive airway pressure (NCPAP) ventilator, are widely used and attributed to the growth and survival of infants and support respiratory failure in NICUs [7]. The use of these treatments could be accompanied by central line-associated bloodstream infection and ventilator-associated pneumonia, reported as healthcare quality indicators regarding HAIs, and are leading causes of morbidity and mortality in the NICU [8].

Effective infection control education is critical in preventing HAIs and reducing their incidence [9], especially in NICUs, which require nurses with extensive experience and proficiency in caring for high-risk newborns to manage their treatment efficiently [10]. Simulation-based education (SBE) places learners in a realistic patient care environment where they can safely practice decision-making and provide care without causing any harm [11]. SBE is also gaining considerable attention because of its capacity to simulate diverse scenarios, including those not frequently encountered in clinical practice [12]. As a new method of providing simulation learning, virtual reality (VR) simulation combines digital learning and simulation to create a unique, three-dimensional, interactive experience that helps users believe they are in an actual healthcare environment [13]. Simulation education utilizing VR provides learners with immersive learning experiences; enhances their learning outcomes [14] and problem-solving abilities by helping them better recall information through vivid scenarios [15]; and positively impacts concentration, commitment, and confidence in achieving learning goals [16,17]. To efficiently leverage these VR simulation training effects, users must be provided with the experience of presence, making the virtual world feel such as the real world and inducing empathy, allowing emotional involvement toward the characters’ emotions in the video [18]. Hence, presence and empathy significantly contribute to the positive effects of VR-based simulation learning [19].

Confidence in nursing was increased among nurses through a VR-based learning intervention involving patients on ventilators [20] and among nursing students through repetitive learning for core nursing skills [21]. A VR program related to infection control in NICU increased confidence in infection control [22]; a hybrid learning program related to nursing children with asthma, combining lectures and high-fidelity simulation increased relevant knowledge, self-confidence, and clinical performance [23]; and a VR 360-degree intravenous infusion training program significantly increased nursing students’ empathy score [24]. Furthermore, VR simulation-based transfusion training increased presence and confidence through easy immersion in the simulated situation, and an increase in nursing students’ knowledge and performance after receiving VR simulation education related to indwelling catheterization [11], cardiopulmonary resuscitation [25], and post-appendectomy care of children [26] was observed. These studies confirm the positive effects of VR simulation programs on improving learners’ knowledge, confidence in nursing practice, and clinical skills.

However, most VR simulation studies have been conducted with nursing students, and few studies involve practicing nurses. Although some studies on neonatal care exist [22,27], they primarily targeted nursing students, with limited research involving NICU nurses.

### 1.1. Study Aims

This study aimed to develop and implement a VR simulation for advanced infection control education in NICUs (VR_AICENICU); assess its effectiveness in enhancing NICU nurses’ knowledge and confidence in infection control; and evaluate nurses’ levels of presence, empathy, and satisfaction associated with the program.

### 1.2. Research Hypotheses

**Hypothesis** **1.**  *The experimental group exposed to the VR_AICENICU program will score higher on “knowledge of infection control” in the post-intervention test than the control group, who have not been exposed to the VR_AICENICU program.*

**Hypothesis** **2.**  *The experimental group will score higher on “confidence in infection control” in the post-intervention test than the control group.*

## 2. Materials and Methods

### 2.1. Study Design

This study used a non-equivalent control group pretest-post-test design to identify the VR_AICENICU program’s effects on infection control knowledge, confidence, presence, empathy, and program satisfaction in NICU nurses.

### 2.2. Participants

This study included NICU nurses working in two general hospitals. Both hospitals have similar numbers of beds, nurse staffing, and patient characteristics, but they are located in different regions. Participants were recruited at two geographically distant sites to prevent contamination between the groups. The experimental group was from G University Hospital in J City and the control group was from CG University Hospital in C City. The inclusion criteria were nurses working in patient care in the NICU. The minimum required sample size was calculated to be 37 using the G * power 3.1.9 program, assuming an effect size (f) set of 0.55, a significance level (α) of 0.05, and a power (1 − β) of 0.90. A large effect size was selected based on comparable previous studies, which reported effect sizes ranging from 0.40 to 0.68 [22,28]. Considering a potential dropout rate of 10%, 40 NICU nurses were recruited, with 20 nurses each in the experimental and control group. There were no dropouts during the program.

### 2.3. Research Tools 

#### 2.3.1. General Characteristics

Participants’ general characteristics included age, sex, education level, marital status, clinical career, NICU work experience, position, prior experience with NICU infection control education, and prior experience with VR-related education.

#### 2.3.2. Infection Control Knowledge

Infection control knowledge was measured using the knowledge-related items excerpted from the ‘’high-risk neonatal infection control competency scale’’ developed by Yu et al. [29] for NICU nurses. The excluded subscale items were “skin care”, “lactation management”, and “environmental management”, not related to the VR_AICENICU program. Twenty-three items were included from the subscales of “basic infection control”, “high-risk medication management”, and “invasive procedure and equipment management” (except for two items related to the umbilical cord catheter). Participants rated each item as “correct”, “incorrect”, or “don’t know”, and each item was scored on a binary scale of 1 (“correct”) and 0 (“incorrect or don’t know”), with a higher total score indicating a higher level of infection control knowledge. The internal consistency reliability (KR-20) of the scale was 0.67 in Yu et al. [29] and 0.37 in this study.

#### 2.3.3. Confidence in Infection Control

Confidence in infection control was measured using the items of the “performance” domain of the high-risk neonatal infection control competency scale developed by Yu et al. [29] for NICU nurses. Twenty items were taken from the subscales of “basic infection control”, “high-risk medication management”, and “invasive procedure and equipment management”, except for one item related to tracheostomy tube replacement not performed by nurses. Participants rated each item on a four-point Likert scale (1 = “strongly disagree”, 4 = “strongly agree”), with a higher total score indicating greater confidence in infection control. The internal consistency reliability (Cronbach’s α) of the scale was 0.95 in Yu et al. [29] and 0.82 in this study.

#### 2.3.4. Presence

Presence was measured using the ‘’presence scale”, with 19 items, developed by Chung and Yang [30] for 3D image evaluation; permission was obtained from the developers, and modifications were made to suit the NICU setting. The revised items were reviewed by a professor of child nursing, a NICU nursing manager, and a senior nurse. The scale comprises four subscales: spatial involvement (seven items), temporal involvement (four items), dynamic immersion (five items), and realistic immersion (three items). Participants rated each item on a five-point Likert scale (1 = “strongly disagree”, 5 = “strongly agree”), with a higher total score indicating greater presence. The Cronbach’s α values of the four subscales were 0.92, 0.89, 0.81, and 0.86, respectively, during scale development, and 0.81, 0.54, 0.49, and 0.56, respectively, in this study. The overall Cronbach’s α for the scale was calculated to be 0.84, indicating good internal consistency.

#### 2.3.5. Empathy

Empathy was measured using the “VR-related empathy scale” developed by Oh [18], with item modifications to suit the NICU setting. For example, the item “I felt the same emotions as did the people in the VR video” was rephrased as “I felt the emotions of the newborn in the VR video on hearing their reactions (crying, pain, etc.)”. Participants rated each item on a five-point Likert scale (1 = “strongly disagree”, 4 = “strongly agree”), with a higher total score indicating greater empathy. The Cronbach’s α of the scale was 0.87 in Oh [18] and 0.89 in this study, indicating good internal consistency.

#### 2.3.6. Program Satisfaction

Program satisfaction was measured using the three-item VR program satisfaction scale developed by Yu et al. [31]. Participants rated each item on a five-point Likert scale (1 = “strongly disagree”, 5 = “strongly agree”), with a higher total score indicating greater program satisfaction. The Cronbach’s α of the scale was 0.81 in Yu et al. [31] and 0.84 in this study, indicating good internal consistency. Furthermore, content analysis was conducted on the VR simulation experience, and its applicability to education was discussed during the debriefing stage. 

### 2.4. VR_AICENICU Program Development

The VR_AICENICU program was developed as follows.

#### 2.4.1. Step 1: Validation of the Infection Control and Development Procedures

Three infection control scenarios were selected for VR simulation: catheter-related bloodstream infection (CRBSI), infection-prone intralipid infusion, and ventilator-associated infection, which are healthcare quality indicators for infection control. These scenarios involve procedures for preventing CRBSI at the catheter insertion site for drug injection in high-risk newborns hospitalized for an extended period, managing intralipid infusion vulnerable to contamination when left at room temperature for a prolonged period or infused in portions, managing the respiratory circuit for a noninvasive NCPAP ventilator, performing gastric decompression to prevent gastric juice reflux, and preventing skin damage from nose mask compression. NICU nurses and doctors must be familiar with these measures, which are infection control techniques and nursing procedures.

The content validity of the statements and procedures of the scenarios was evaluated by a seven-member expert panel comprising a nursing professor, a NICU nurse manager, three experienced nurses with at least 10 years of clinical experience, a teaching nurse with a master’s degree in child nursing and VR simulation development experience, and a nurse specializing in nosocomial infection. The Delphi technique was used to gather experts’ opinions on each procedure or statement using a four-point Likert scale (1 = “strongly disagree”, 2 = “disagree”, 3 = “agree”, 4 = “strongly disagree”).

The panel members were also requested to provide their opinions on related topics through open-ended questions. An item-level content validity index (I-CVI) was used to determine those who rated 3 or 4 on each item, and the items with an I-CVI of 0.80 or higher were used without modifications, while those lower than 0.80 were revised three times. Expert opinions were documented in an open-ended format. The final versions of the three scenarios included 18 steps related to CRBSI, 15 related to intralipid infusion, and 13 related to noninvasive ventilator infection control.

#### 2.4.2. Step 2: Modeling and Construction for VR Implementation

**Figure 1:**
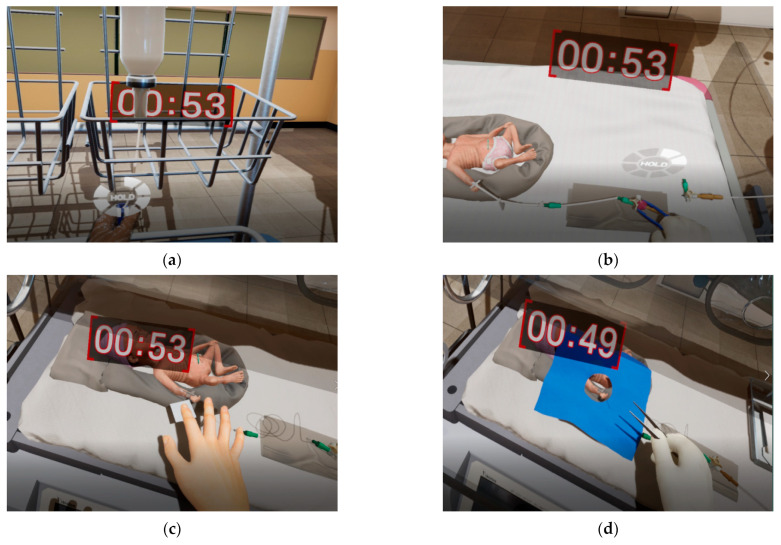

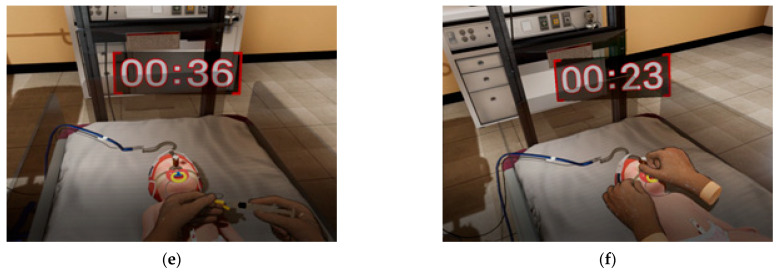
Images from the VR_AICENICU Program. VR_AICENICU = virtual reality simulation for advanced infection control education in neonatal intensive care units. (**a**) Scenario 1: Prepare an intralipid solution; (**b**) Scenario 1: Disinfect the connector before connecting the fluid; (**c**) Scenario 2: Removal of skin residue from the peripherally inserted central catheter (PICC); (**d**) Scenario 2: Disinfection of the PICC insertion site; (**e**) Scenario 3: Decompression to prevent gastric reflux; (**f**) Scenario 3: Skin care around the nose mask.

To implement the three scenario modules as a VR simulation program, a VR environment mimicking the NICU setting was created using equipment and tools, including an automatic faucet, a radiant warmer, an incubator with a cover, a treatment table, an infusion pump, a ventilator, a biohazard waste bin, a preterm infant mannequin, and various medicines and disinfectants. A research assistant and a neonatal education nurse conducted demonstrations following the scenario steps. Based on the demonstration recording, the VR program was completed through the 3D modeling and animation production process. Hand-tracking technology enables the user to select objects through hand movement. Audio sources replicating sounds such as a baby’s cry and alarms commonly heard in a NICU setting were added to provide a more realistic, engaging, and immersive experience (Figure 1).

#### 2.4.3. Step 3: VR Program Equipment and Software

The equipment used to produce the VR simulation program included a VR kit containing an EliteDesk 800 G4 laptop computer, a Vive Pro Full-Kit Head Mounted Display (HMD) and sensor (HTC VIVE, Bellevue, WA, USA), and a LEAP Motion Controller^TM^ (Ultraleap, Silicon Valley, CA, USA) hand-tracking device with a VR Developer Mount. Additionally, one large TV monitor was employed to allow multiple users to view the program concurrently. The VR simulation program software was commissioned by a VR manufacturer (SAMWOOimmersion Co., Ltd., Busan, Republic of Korea) based on prerecorded images and video recordings of the three simulation scenarios.

### 2.5. Study Procedures

The experimental group underwent pre-survey, experimental treatment, and post-survey from 1–20 February 2022. The control group underwent pre-survey and post-survey from 7–30 July 2022, in accordance with the approval date from the Institutional Review Board. The details of the procedure are described below.

#### 2.5.1. Pre-Survey

The pre-survey, which collected information on general characteristics, infection control knowledge, and confidence in infection control, was conducted in early February for the experimental group and in early July for the control group. The questionnaire was distributed and collected by the researcher in person.

#### 2.5.2. Experimental Treatment

The 20 participants in the experimental group were divided into small groups of three to five members considering their work schedule, and the program was conducted from 10–15 February 2022. Each program session comprised three stages: pre-briefing, VR simulation, and debriefing. The pre-briefing stage, which lasted 20 min, was divided into three parts: infection control principles’ overview and scenarios presentation, VR simulation practice environment presentation, and HMD and LEAP motion controller use demonstration along with precautions. The VR simulation lasted 10–15 min per scenario. During a 20-min debriefing stage, users discussed their VR simulation experience and its applicability to education. The program lasted 80 min in total (Table 1). As for the control group, participants were given a handout on infection control for NICU and received conventional lecture-centered education.

#### 2.5.3. Post-Survey

The experimental group was instructed to complete the post-survey immediately after the program ended. The control group was given the post-survey two weeks after the pre-survey to prevent the testing effect and maintain internal validity.

### 2.6. Data Analysis 

Data analysis was performed using the SPSS/WIN 25.0 program (IBM Corp., Armonk, NY, USA). General characteristics were analyzed using frequency, percentage, mean, and standard deviation. The homogeneity between the experimental and control groups was tested by performing the chi-square test, independent *t*-test, and non-parametric Mann–Whitney test. The study variables’ normality was examined by performing the Shapiro–Wilk test. Intergroup differences in infection control knowledge and performance confidence were analyzed by performing Wilcoxon’s signed-rank and Mann–Whitney tests. The presence, empathy, and program satisfaction of the experimental group were analyzed using mean and standard deviation.

### 2.7. Ethical Considerations 

Before data collection, approval was obtained from the Institutional Review Board of the two hospitals where the experimental and control groups were recruited (GNUH 2021-09-024-002 and GNUCH 2022-05-023-001). The researcher explained the study purpose and procedure to the participants, the anonymity and confidentiality in handling the data, and their right to withdraw from participation at any time and obtained their written consent. Each completed questionnaire was placed in an envelope and collected directly by the researcher. Each participant was compensated with a gift card worth approximately 30 USD, along with snacks. The control group was informed of the possibility of participating in the VR simulation program and receiving infection control education materials. 

## 3. Results

### 3.1. Homogeneity Testing of General Characteristics and Study Variables

The homogeneity testing between the experimental and control groups, conducted before program implementation, confirmed homogeneity in the general characteristics except for education level (χ^2^ = 9.23, *p =* 0.002). Regarding study variables, homogeneity was confirmed only in infection control knowledge (Z = −1.09, *p* = 0.314; Table 2).

### 3.2. Program Effectiveness Testing

#### 3.2.1. Hypothesis 1

The post-test infection control knowledge score increased significantly compared to the pre-test score only in the experimental group (Z = −2.95, *p* = 0.003), with the control group showing no significant change (Z = −1.26, *p* = 0.210). However, as no intergroup differences were observed in the post-hoc test score (Z = −1.88, *p* = 0.063), hypothesis 1 was rejected. Regarding individual items of infection control knowledge, while no significant intergroup differences were observed in invasive procedures, instrument management, and medication management, the mean score for basic infection control was significantly higher in the experimental group than in the control group (Z = −2.00, *p* = 0.045; Table 3). 

#### 3.2.2. Hypothesis 2

The post-test score for confidence in infection control increased significantly only in the experimental group (Z = −2.27, *p* = 0.024), with no significant change observed in the control group (Z = −0.97, *p* = 0.360). The experimental group scored significantly higher for confidence in infection control than the control group (Z = −2.92, *p* = 0.004), supporting Hypothesis 2. Considering individual items of confidence in infection control, the experimental group scored significantly higher than the control group in basic infection control (Z = −2.95, *p* = 0.003), and significant intergroup differences were observed in invasive procedures, instrument management, and medication management (Table 3).

### 3.3. VR Simulation Program-Related Presence, Empathy, and Satisfaction

The experimental group was examined for presence, empathy, and program satisfaction related to the VR simulation program after the intervention. The overall mean score was 4.39 ± 0.36 for presence, 4.33 ± 0.75 for empathy, and 4.90 ± 0.31 for program satisfaction (Table 4).

## 4. Discussion

Accurate understanding and rigorous implementation of effective infection prevention measures in nursing are crucial to enable a nurse to minimize HAIs [32]. NICU nurses perform several tasks and play a crucial role in newborn care; they are in consistent contact with hospitalized infants and medical staff [33]. Newborn care is performed in a highly complex and dynamic environment, and the prognosis of newborns significantly varies depending on the care quality [34]. Nurses with limited experience could inevitably feel anxious about newborn care owing to limited skills and ability to predict outcomes [35]. Substantially improving practical skills in actual nursing settings through simple observations in clinical settings alone is challenging as well [36]. This highlights the need for future nursing education innovation wherein students can actively participate and acquire realistic experiences directly applicable to actual clinical settings [14]. Hence, this study developed an advanced infection control education program using VR simulation to overcome the education limitations in clinical settings and strengthen the infection control competency of NICU nurses.

The VR_AICENICU program is designed to help NICU nurses practice three scenarios: high-risk medication infusion management, insertion site care of a preterm infant with a peripherally inserted central catheter (PICC) line, and newborn care on NCPAP. In this process, NICU nurses can apply aseptic techniques when performing high-risk medication insertion and infusion, assessing PICC site infection, preparing items necessary for dressing changes, dressing the PICC insertion site, handling the NCPAP machine, assessing the skin around a nasal mask, and performing respiratory nursing. These procedures can help NICU nurses learn techniques for preventing nosocomial infections related to CRBSI, ventilator-related pneumonia, and skin infections frequent in premature infants. Additionally, to help users practice proper hand hygiene and personal protective equipment use during sterile dressing changes, the execution time for each procedure is set (e.g., 40–60 s for hand hygiene), and the program algorithm is arranged to allow users to progress to the next step only after appropriate execution of the current step. Therefore, users can accurately learn the basic infection control procedures and sequences by repeatedly practicing on a neonatal mannequin in a safe virtual environment even if they make mistakes. 

The scenarios developed in this study correspond to the medications and treatments that nurses use when treating premature infants in a NICU setting. However, to ensure patient safety, novice nurses are not allowed to care for severely ill, high-risk newborns or premature infants without relevant treatment experience. Therefore, this study’s educational course provides an appropriate training program for novice nurses to care for premature infants. The satisfaction score obtained in this study was higher than scores achieved by nursing students [22] exposed to VR simulation scenarios with high-risk newborns. Furthermore, participants evaluated the VR_AICENICU program as suitable for new nurse education and training with statements such as “The program was a great help for me as a novice nurse” and “It will be a good educational and practice opportunity for the newly hired nurses, who have not had sufficient clinical practice due to COVID-19.” The program was also believed to provide practical education for nurses, as observed through statements such as “Through the VR simulation education, I became more vigilant toward infection control with an increased awareness for hand hygiene and thorough practice of other infection control measures”; “I had the opportunity to familiarize myself with the infection control protocol once more by gaining a clear understanding of the aspects that can be confusing in clinical settings”; and “I believe that the program offers current nurses an opportunity to learn and practice proper infection control procedures and correct any mistakes they tend to make”.

Based on these opinions, the VR_AICENICU program could benefit both introductory education for new nurses and relearning education for experienced nurses. Additionally, the high level of program satisfaction and intention to recommend the program to others suggests that it can become a part of nurse education, providing the empirical foundation for the integration of the VR SBE program. 

Comparing infection control knowledge and performance confidence between NICU nurses who received VR-based simulation education (experimental group) and those who received conventional lecture-centered oriented education (control group) showed that the experimental group scored significantly higher in infection control knowledge. This finding aligned with those of Yoon’s [20] study, which reported higher knowledge scores among novice nurses who received VR simulation education involving patients on ventilators. In the VR_AICENICU program, the experimental group received lectures on basic infection control, central venous catheterization, ventilator care, and high-risk medication management in the pre-briefing phase and practiced the relevant procedures repeatedly in the simulation stage, contributing to improving knowledge and practical skills related to infection control [11]. However, no significant difference was observed in the infection control knowledge scores between the experimental and control groups in the post-test (Hypothesis 1), aligning with Yu et al.’s [22] findings, based on infection-related VR simulation intervention for nursing students and high-fidelity mannequin simulation training regarding infection and pre-eclampsia in maternal-newborn nursing [36]. In this study, specifically, both groups scored equally high in the pre-test (≥21 out of 23 points) and had many years of NICU experience (mean = 3.63 years), which supposedly left no margin for significant improvement in the groups irrespective of intervention. However, a significant intergroup difference was observed in the post-test scores for basic infection control, one of the subscales of infection control knowledge, which could be from the debriefing stage because the experimental group obtained a better understanding of the areas previously confusing in clinical practice.

SBE can induce learning motivation by improving learners’ confidence, the main advantage of applying simulation to education [37]. In this study, the experimental group scored significantly higher than the control group on confidence in infection control practices (Hypothesis 2), aligning with previous studies on VR simulation training programs for nurses and nursing students involving patients receiving mechanical ventilation [20], hospitalization management [38], and home nursing [14], wherein participants’ performance confidence increased after receiving VR simulation training. These findings suggest that VR simulation training contributed to improving participants’ confidence in performing relevant skills. However, the scores for presence and empathy among clinical nurses in this study were relatively higher than the scores of nursing students exposed to VR simulation-based transfusion training [39]. To determine the effects of VR simulation training, we measured nurses’ presence and empathy levels when exposed to the VR_AICENICU program and obtained mean scores of 4.39 and 4.35, respectively. They scored higher for presence than nursing students exposed to VR simulation-based transfusion training, who scored 130.75 (3.45 when converted to a five-point scale) [39]. Participants of the VR_AICENICU program reported a perceived notion of touching real items or drugs in the VR environment and treating patients in a real-life clinical setting.

The VR_AICENICU program was developed using actual demonstrations of the nursing procedures by a NICU training nurse following the planned scenarios, modeling of the demonstrations, and several revisions for program refinement. The final program accurately replicated NICUs’ materials and equipment, such as an incubator and an infusion pump, and included background noises such as monitor alarms, phone ringing, and nurses talking. However, there were technical issues such as functional errors in recognizing nurses’ movements, which sometimes required repeating the exact movement, and loading-related disconnections. These limitations could be addressed by enhancing the LEAP motion sensor sensitivity, an important VR device, and reducing program errors to increase user immersion and presence.

The experimental group had a mean post-test empathy score of 4.33 out of 5 points, showing a higher level of empathy for patients in the VR simulation video than in previous studies using the same empathy scale of 3.80 in a study using VR 360-degree intravenous infusion training for nursing students [24] and 3.16 in a study examining users’ empathy toward a VR advertisement [18]. In this study, participants were experienced NICU nurses who had provided care for high-risk newborns in clinical settings. They showed empathy specifically by patting or comforting a crying infant and providing utmost care when removing a dressing to avoid pain, even in the VR environment. During the debriefing stage, some participants made remarks such as “While experiencing the VR simulation program, I found myself performing nursing care without even realizing it. It felt as if I was directly performing it”. Unlike previous studies on VR simulation programs for learning and practicing nursing techniques, the VR_AICENICU program provides scenarios for NICU nurses to care for high-risk newborns in a realistic NICU setting, allowing for deep immersion in nursing situations and experiencing empathy more vividly. 

This study is significant because it is the first VR simulation-based advanced infection care training program for NICU nurses that includes implementation and assessment. The results provide empirical data for introducing and utilizing VR simulation-based training programs for nurse education and practical training. The VR_AICENICU program can help nurses learn and practice nursing skills, which are challenging to perform on high-risk newborns, by providing realistic experiential learning opportunities in a safe VR environment. It can also help nurses who have temporal constraints due to shiftwork. Since it enables nurses to learn through repeated practice on a virtual patient in a safe virtual environment, it can strengthen nursing competency, improving patient safety.

This study has some limitations. First, since convenience sampling was used to recruit NICU nurses from two general hospitals in two different regions, caution is required when generalizing the results to all NICU nurses. Second, homogeneity in education level between the experimental and control groups could not be established because the NICU nurses from the experimental and control groups were from two different general hospitals. Additionally, owing to the limited number of NICU nurses, all nurses except managers were enrolled in this study. Therefore, differences in participants’ education level and nursing experience based on the severity level of high-risk newborns could not be controlled for in the baseline homogeneity test.

## 5. Conclusions

Participants who received the VR_AICENICU program showed increased infection control knowledge and performance confidence, and they attained high scores for presence in the VR environment and empathy for patients. These results verify the feasibility of the VR_AICENICU program as an effective infection control training tool for new and experienced nurses. Future studies should apply the VR_AICENICU program to different severity grades of NICU patients and develop additional VR programs, given the positive outcomes, to enhance nursing competencies in other areas and test their effectiveness.

## Figures and Tables

**Table 1:** Contents and procedures of the VR_AICENICU program.

Stage	Contents		Time (Min)
Stage 1. Pre-briefing	Preparation	Lecture on infection control guidelines	20
	Orientation	Presentation of the scenarios	
		VR-use-related demonstrations and precautions	
Stage 2. VR Simulation	Scenario 1. Infection-prone intralipid infusion A high-risk newborn should be infused with intralipid for nutrition supply. The newborn has a PICC in the right arm and is currently connected to total parenteral nutrition. The user should additionally administer intralipid through a three-way catheter. The prescription infusion rate is 1 cc/h every 12 h.		15
	Scenario 2. Insertion site care of a preterm infant with a PICC line The injection site dressing appears loose, and a slight skin flare is observed in a 700 g premature newborn receiving intravenous nutrition through a PICC line. To prevent infection, aseptic techniques must be strictly followed when changing the dressing.		15
	Scenario 3. Care of a newborn on NCPAP A 2700 g high-risk newborn is under respiratory support with NCPAP. The user must perform routine checks on the proper functioning of the respiratory equipment, humidification temperature, skin condition around the nasal mask, and gastroesophageal reflux. (NCPAP setting: FiO_2_ 0.4, Pressure 5 cmH_2_O, Flow 8 L/min, Humidifier: 37 °C)		10
Stage 3. Debriefing	How did the last simulation go? How do you feel now that the simulation is over? How did you feel about the simulated situation? How was the preterm infant you met in the VR environment? Were there any difficulties during the VR simulation? How is he (infant) different from what you experienced in the VR simulation today?		20
Total			80

Note. VR_ AICENICU = virtual reality simulation for advanced infection control education in neonatal intensive care units; NCPAP = nasal continuous positive airway pressure; PICC = peripherally inserted central catheter.

**Table 2:** General characteristics, study variables, and homogeneity (*N* = 40).

Characteristic	Category	Total (%)	Group		χ^2^/t/Z ^†^	*p*
			Control Group (*n* = 20)	Experimental Group (*n* = 20)		
Educational level	3-year college	13 (32.5)	11 (55.0)	2 (10.0)	9.23	0.002 **
	4-year college	27 (67.5)	9 (45.0)	18 (90.0)		
Infection control training	Yes	25 (62.5)	12 (60.0)	13 (65.0)	0.11	0.744
	No	15 (37.5)	8 (40.0)	7 (35.0)		
VR experience *	Yes	1 (2.5)	-	1 (5.0)		1.000
	No	39 (97.5)	20 (100.0)	19 (95.0)		
Infection control knowledge	Basic infection control	9.60 ± 0.78	9.35 ± 0.99	9.85 ± 0.37	−1.89	0.142
	Invasive procedure and equipment management	4.65 ± 0.66	4.65 ± 0.59	4.65 ± 0.75	−0.31	0.820
	High-risk medication management	7.70 ± 0.52	7.70 ± 0.47	7.70 ± 0.57	−0.24	0.862
	Total	21.95 ± 1.15	21.70 ± 1.34	22.20 ± 0.89	−1.09	0.314
Confidence in infection control	Basic infection control	3.77 ± 0.25	3.61 ± 0.26	3.93 ± 0.10	−4.13	<0.001 ***
	Invasive procedure and equipment management	4.00 ± 0.00	4.00 ± 0.00	4.00 ± 0.00	0.00	1.000
	High-risk medication management	3.91 ± 0.17	3.84 ± 0.20	3.99 ± 0.06	−3.12	0.013 **
	Total	3.87 ± 0.16	3.77 ± 0.17	3.97 ± 0.05	−4.11	<0.001 ***

Note. * Fisher’s exact test, ^†^ Mann–Whitney test, NICU = neonatal intensive care unit, VR = virtual reality, ** *p* < 0.05, *** *p* < 0.001.

**Table 3:** Differences in infection control knowledge and self-confidence for infection control performance between groups (*N* = 40).

Variable	Category	Group	Pre-Test	Post-Test	Difference between Time	Program Effect
			Mean ± SD	Mean ± SD	Z (*p*) *	Z (*p*) ^†^
Infection control knowledge	Basic infection control	Exp.	9.35 ± 0.49	9.95 ± 0.22	−2.33 (0.020)	−2.00 (0.045 **)
		Cont.	9.85 ± 0.39	9.85 ± 0.37	0.00 (1.000)	
	Invasive procedure and equipment management	Exp.	4.65 ± 0.59	5.00 ± 0.00	−2.33 (0.020)	−0.88 (0.495)
		Cont.	4.65 ± 0.75	4.90 ± 0.31	−1.41 (0.157)	
	High-risk medication management	Exp.	7.70 ± 0.47	7.95 ± 0.22	−1.89 (0.059)	−1.16 (0.327)
		Cont.	7.70 ± 0.57	7.75 ± 0.44	−0.30 (0.763)	
	Total	Exp.	21.70 ± 1.34	22.90 ± 0.31	−2.95 (0.003)	−1.86 (0.063)
		Cont.	22.20 ± 0.89	22.50 ± 0.61	−1.26 (.210)	
Confidence for infection control	Basic infection control	Exp.	3.61 ± 0.26	3.86 ± 0.21	−2.24 (.015)	−2.95 (0.003 **)
		Cont.	3.93 ± 0.10	3.95 ± 0.11	–0.79 (0.430)	
	Invasive procedure and equipment management	Exp.	4.00 ± 0.00	3.99 ± 0.06	−1.00 (0.317)	−0.04 (0.989)
		Cont.	4.00 ± 0.00	3.98 ± 0.11	−1.00 (0.317)	
	High-risk medication management	Exp.	3.84 ± 0.20	3.91 ± 0.19	−1.58 (0.115)	−1.37 (0.277)
		Cont.	3.99 ± 0.06	4.00 ± 0.00	−1.00 (0.317)	
	Total	Exp.	3.77 ± 0.17	3.90 ± 0.16	−2.27 (0.024)	−2.92 (0.004 **)
		Cont.	3.97 ± 0.05	3.97 ± 0.07	−0.92 (0.360)	

Note. * Wilcoxon’s signed-ranks test, ^†^ Mann–Whitney test, Exp. = experimental group, Cont. = control group, ** *p* < 0.05.

**Table 4:** Presence, empathy, and program satisfaction (*N* = 20).

Variable	Sub-Item	Range	Mean ± SD
Presence	Spatial involvement	1–5	4.44 ± 0.45
	Temporal involvement	1–5	4.08 ± 0.62
	Dynamic immersion	1–5	4.62 ± 0.35
	Realistic immersion	1–5	4.33 ± 0.46
	Total		4.39 ± 0.36
Empathy		1–5	4.33 ± 0.75
Program satisfaction	1. I think this program will help me to work as a neonatal intensive care unit nurse	1–5	4.90 ± 0.31
	2. I would recommend this program to other nurses	1–5	4.90 ± 0.31
	3. I think this education program is necessary for the nurse education curriculum	1–5	4.90 ± 0.31
	Total	1–5	4.90 ± 0.31

## Data Availability

Data is unavailable due to privacy or ethical restrictions.
